# In Defence of informed consent for health record research - why arguments from ‘easy rescue’, ‘no harm’ and ‘consent bias’ fail

**DOI:** 10.1186/s12910-020-00519-w

**Published:** 2020-08-20

**Authors:** Thomas Ploug

**Affiliations:** Aalborg University, Centre for Applied Ethics and Philosophy of Science, Department of Communication and Psychology, A C Meyers Vænge 15, 2450 Copenhagen, SV Denmark

**Keywords:** Informed consent, Health data, Duty of easy rescue, Harm, Medicalization, Privacy, Trust, Stigmatization, Equality in health, Solidarity, Consent bias

## Abstract

**Background:**

Health data holds great potential for improved treatments. Big data research and machine learning models have been shown to hold great promise for improved diagnostics and treatment planning. The potential is tied, however, to the availability of personal health data. In recent years, it has been argued that data from health records should be available for health research, and that individuals have a duty to make the data available for such research. A central point of debate is whether such secondary use of health data requires informed consent.

**Main body:**

In response to recent writings this paper argues that a requirement of informed consent for health record research must be upheld. It does so by exploring different contrasting notions of the duty of easy rescue and arguing that none of them entail a *perfect* duty to participate in health record research. In part because the costs of participation cannot be limited to 1) the threat of privacy breaches, but includes 2) the risk of reduced trust and 3) suboptimal treatment, 4) stigmatization and 5) medicalisation, 6) further stratification of solidarity and 7) increased inequality in access to treatment and medicine. And finally, it defends the requirement of informed consent by arguing that the mere possibility of consent bias provides a rather weak reason for making research participation mandatory, and that there are strong, independent reasons for making.

**Conclusion:**

Arguments from the duty of easy rescue in combination with claims about little risk of harm and potential consent bias fail to establish not only a *perfect* duty to participate in health record research, but also that participation in such research should be mandatory. On the contrary, an analysis of these arguments indicates that the duty to participate in research is most adequately construed as an *imperfect* duty, and reveals a number of strong reasons for insisting that participation in health records research is based on informed consent.

## Background

For decades the Helsinki Declaration’s dictum that the interests of an individual must prevail over the interests of society has been one of the guiding principles for medical research, and informed consent has been the cornerstone of protecting research participants [1]. In the last decades, a number of writers have defended the idea that medical research based on data from electronic patient records do not require informed consent [2, 3].

This idea is also pursued in a comprehensive argument in a fairly recent paper on health record research by Mann, Savulescu and Sahakian [4]. The authors argue 1) that the duty of easy rescue entails a moral obligation to participate in health records research since 2) the potential benefits are significant and the harm associated with such research is first and foremost the risk of a loss of privacy, which is minimal. They further argue 3) that consent requirements pose a significant problem to health records research because it may lead to consent bias that in turn may have harmful consequences. The combination of a duty to participate in health record research and the consent bias problem lends itself to the conclusion that participation in health record research should be mandatory in cases where there are significant benefits and only insignificant harms such as the risk of a loss of privacy. In line with this, the authors suggest that 4a) ‘the default position of research ethics committees should be to grant access to minimally risky uses of patient data without the need for consent’, and 4b) that in cases with greater than minimal risk, ‘a national or state-level EHR research authority be established and invested with the power to grant research exemptions for the requirement of informed consent’.

This article counters each of the claims underlying the asserted exceptionalism of health record research. More specifically, it …

1. Explores different contrasting notions of the duty of easy rescue and argues that none of them entail a *perfect* duty to participate in health record research, and
2. Argues that the costs of participation cannot be limited to the threat of privacy breaches, but includes the risk of reduced trust and suboptimal treatment, stigmatization and medicalisation, further stratification of solidarity and increased inequality in access to treatment and medicine, and
3. Argues that the mere possibility of consent bias provides a rather weak reason for making research participation mandatory, and
4. Argues that that there are strong reasons for insisting that participation in health records research is based on informed consent, including that research ethics committes cannot adequately protect individuals’ values and interests, and that informed consent fosters trust in research/researchers

In recent years there has been considerable debate on different models of informed consent. A range of different models and solutions have been developed and debated in relation to different types of research, including specific and broad consent models, opt-out solutions, and most recently the dynamic consent and the meta consent models [5–23]. Although the analysis in this article certainly is of relevance for these debates, it considers the more fundamental question of why health record research should at all be based on informed consent. The article shows why a requirement of informed consent to such research is indispensable – not that this requirement can only be satisfied by one particular model of informed consent. It may very well be the case – as argued in the concluding section – that certain models of consent that put less of a strain on research and researchers, may be the appropriate implementations of a requirement of informed consent. Yet, how to balance the many different interests in the choice of a model of informed consent is a wholly separate question. It follows, however, that informed consent for research participation in this article is interpreted rather minimally. Thus, it will throughout simply denote the proces whereby an individual is provided with the choice of whether or not to participate in research on the basis of having received adequate information and without being under undue influence.

The article specifically concerns the question of informed consent for health record research. The analysis applies, however, to the wider context of health care research as many of the considerations and arguments laid out are not specific to health record research, but will hold for any type of health care research.

## Discussion

### Different conceptions of the duty of easy rescue

Any attempt at showing that the duty of easy rescue has implications for participation in research should consider the diversity in views on the content of this duty. Two contrasting notions at each end of the spectrum are Peter Singer’s and Patricia Greenspan’s [24–28].

In Singer’s definition the duty of easy rescue is a moral obligation we have “if it is in our power to prevent something bad from happening, without thereby sacrificing anything of comparable moral importance” [29]. Or, to use his famous application of the duty, if I am walking down the road and see a child drowning in a shallow pond, then I have a duty to wade in and save the child, although I may get muddy trousers. Although the case of the drowning child suggests that duty of easy rescue could be limited to situations only where life is endangered, Singer’s definition does not limit the duty to such cases. On the contrary, he argues that the duty also implies a more general duty to aid people by donating to charity efforts to provide food, shelter and medical care for refugees.

Singer’s duty of easy rescue has a number of distinctive features. Firstly, it does not ascribe moral significance to proximity and personal relations. It is not limited to rescuing people with whom we are close in time and space or with whom we have a special relationship. It extends beyond family and friendships, and it extends beyond the borders of nation-states and generations. The duty concerns the prevention of harm to anyone, anywhere and anytime. Secondly, it is a duty that obtains in all situations in which action is *sufficient* to prevent harm. The moral obligation to wade in and save the drowning child is not limited by whether or not there are other bystanders that could also save the child and perhaps could be argued to have a stronger moral obligation to do so. Thirdly, it is a duty to maximise the prevention of harm. Singer explicitly states that it is a duty to prevent as much suffering as possible without sacrificing anything morally comparable. This feature solves the apparent problem of how to prioritise between the many people whose suffering one may prevent or alleviate at any given point in time. Rather paradoxically, however, this also means that the duty of easy rescue arguably may require us to ignore the drowning child in the shallow pond if more harm can be prevented by doing so.

A contrasting view of the duty of easy rescue is presented by Patricia Greenspan [30]. In her interpretation, the duty of easy rescue is a duty to act whenever we pass by victims of accident and there is no one else available to help, i.e. when help is *necessary*. Greenspan take this duty to be *perfect* in the Kantian sense that it spells out exactly what is owed to others. It is a duty to instantly attempt the rescue of the drowning child. As such the duty of rescue must be distinguished from two other duties. First, a more general, but *imperfect* duty to aid or care for people in need. We have, Greenspan contends, such a broader duty to aid others, but it is a duty that does not entail specifically to whom and how much aid is owed. In acting on this duty we have ‘moral leeway’ to give priority to, for instance, pain over death, women over men etc. Second, the state’s duty of rescue. According to Greenspan, the state has a duty of rescue towards people in need that are not ‘nearby’ as seen from the individual’s perspective. The state’s duty of rescue is ultimately to be explained by a combination of the citizens’ imperfect duty of aid and care and the state’s power and resources that make it more effective in rescuing and aiding distant people. The citizens have an obligation to aid and care for people in need, and the state may effectively coordinate the individual citizens’ efforts and reach people across time and space. However, by in this way transferring their duty of aid and care to the state, citizens must accept that this imperfect duty becomes less imperfect. Thus, the state may to some extent determine the citizens’ contributions and its beneficiaries in order to satisfy its duty of rescue, i.e. citizens will not have direct control over whether their contributions are directed at certain subgroups in society or at particular purposes. Greenspan contends that our derivative duties are satisfiable first and foremost via taxation. Requiring citizens to provide labour and other non-monetary contributions raise harder issues of autonomy, but national service may be defensible if it leaves room for individual choice, i.e. if it is short-term and is not for a specific form of service, e.g. military service.

Greenspan’s position is further explored in a later section. For present purposes it should be noted that the duty of easy rescue so construed is in contrast to Singer’s notion in at least three respects. First, it is different by implying that proximity matters. The individual’s duty is limited to emergencies where we pass by the victims of accident. It does not extend to geographically distant people or future generations. Secondly, it is different by being limited to situations where our actions are *necessary* for the rescue of people. Third and finally, it is different from Singer’s duty of easy rescue by being an *absolute* duty to rescue a person in need. It is not a duty to maximise the prevention of harm. Thus, the duty of easy rescue is a duty to rescue a person also in situations where more harm may be prevented by acting in ways that entail foregoing the rescue of the drowning child. Interestingly, writers on the “rule of rescue” – the duty of doctors to give priority to critically ill patients they are faced with – consider this feature essential to this rule [31–33].

### Singer’s duty of easy rescue and health record research

Inspired by Singer’s famous example of the drowning child, Mann et al. define the duty of easy rescue thus: “When the cost to X of performing some action, G, is small, and the benefit to Y is large, then X ought to G”. This, however, is not a duty of easy rescue. This is a duty of low-cost beneficence. Yet in both Singer’s and Greenspan’s interpretation, the duty of easy rescue is a duty to prevent harm. For good reason. While it is intuitively clear that I have a duty to wade in and save a child I see drowning in a shallow pond, it is a far less plausible assertion that I have a duty to buy an inflatable unicorn to a child I see bathing in a pond even if this would be an insignificant cost to me, but a great benefit to the child.

For present purposes let us therefore consider the duty of easy rescue defined as a duty to prevent harm. Does this duty entail a duty to participate in health record research?

### Does giving data for health record research “prevent something bad from happening”?

On Singer’s account the duty of easy rescue is a duty to maximise the prevention of harm. If participation in health record research should be entailed by a duty of easy rescue, it must therefore be a way of maximising the prevention of harm. That participation in such research would maximise the prevention of harm is questionable for at least two reasons [34].

First and foremost, it is questionable because conducting health record research does not necessarily lead to the prevention of harm. The purpose of health record research is not always to prevent any significant harm. The purpose may be to confirm existing knowledge, and successful research would therefore make no difference for existing health care practices. Also, as all other types of research, health record research may have negative results, i.e. it may lead to no new findings that can be used for the prevention of harm. Furthermore, there is a lengthy, complex and unpredictable path from scientific discovery to actual health care. Developing new interventions and documenting safety, efficiency and cost-efficiency to the proper authorities as well as ascertaining attention and support from commercial and non-commercial stakeholders and decision-makers are just a few of the many preconditions of moving from research to actual health care and the prevention of harm. Health care research that could and would prevent serious harm if implemented in actual health care, may never be implemented. Moreover, research may for many reasons become obsolete before implementation in health care. Finally, even if health care research eventually translates into actual health care, it may be available to patients on conditions that will limit the prevention of harm (see below on inequality of access to medicine). Thus, health care research may lead to new or improved interventions that due to its price may only be accessible to very few patients.

Secondly, it is also questionable because there are alternative and more direct ways of preventing harm than to participate in health care research. One may provide financial support to relief efforts directed at starving people or victims of natural disasters around the world. One may become a blood, bone marrow, stem cell or kidney donor in order to save the lives of ill people. One may donate money to hospices in order to ease the suffering of people in the last stages of their lives [34]. These are all actions that arguably will result in the prevention of harm, i.e. death and suffering, with greater certainty than participation in health care research as such. They give us reason to suspect that there will always be an alternative to participation in health care research that will be preventing harm equally or more. If so, there is no basis for giving priority to research participation over alternatives in the attempt to maximise the prevention of harm. Metaphorically speaking, even if the drowning child in Singer’s example is research as such, there still are alternative and effective ways of preventing harm than to participate in research.

The list of alternatives becomes even longer when considering alternatives to participation in health record research specifically. Thus, there are many ways of contributing to health care research. One may enrol in research based on clinical testing and experiments. One may provide tissue samples for biobank research. One may provide various kinds of data from social platforms or wearables to epidemiological research. These are all ways of conducting health care research that may be equally or more effective in preventing harm than health record research. Moreover, they give reason to suspect that there will always be a research participation alternative that will be preventing harm equally or more. If so, there is no basis for giving priority to any single type of research participation in the attempt to maximise the prevention of harm.

### Does giving data for health record research really NOT entail sacrificing anything of moral importance?

While the uncertainty about the effects of health care research and the availability of alternative courses of action suggests that there could be multiple ways of satisfying a duty of easy rescue, it does not show that a Singer style duty of easy rescue does not entail a duty to participate in health record research. This also depends on the cost of these different ways of satisfying the duty of easy rescue. If the net gain from participation in health record research is greater than the net gain from any alternative taking probabilities into account, the duty of easy rescue would entail a duty to participate only if the costs of participation are incomparable to the benefits. On Singer’s account, the duty of easy rescue applies only to situations where rescue may be achieved at incomparable cost to the rescuer. Even if there are no alternative courses of rescuing, the duty to save the drowning child only upholds if it can be done without sacrificing anything of comparable moral worth. That is, even if we take there to be no alternatives to participation in health record research, there only is a duty of easy rescue to participate in such research if it does involve sacrificing anything of comparable moral worth. One way of severing the link between the duty of easy rescue and the duty to participate in health record research is therefore to show that potential costs of such participation are significant.

#### The costs of health record research 1: privacy breaches

Although Mann et al. *en passant* mentions the possibility of other harms, they only explore the risk of privacy breaches and the harms following such breaches. Based on figures from the U.S. Department of Health and Human Services Office for Civil Rights on privacy breaches affecting 500 individuals or more, they provide statistics evidencing that in the US there is a 0,02% chance of electronic health record breach per person per year by health care providers [4]. They add that many of these breaches stem from primary care and not from research, that not all electronic patient records contain sensitive information, and that only some of the breached records will be used for nefarious purposes. In conclusion they state “We suspect that the vast majority of proposed EHR research carries only minimal risk of harm”. Limiting the costs of participation in health records research simply to privacy breaches where data is used for nefarious purposes is inadequate for two reasons. First of all, it is inadequate because the costs of privacy breaches cannot be limited to the use of data for nefarious purposes, but could also derive from the privacy breaches per se. A loss of informational privacy – i.e. that A gets access to information considered private by B – may cause harm by revealing facts that B consider irrelevant, incomplete, misleading, as belonging to the past etc. This harm – the negative mental states following such revelations, e.g. worry, concern, shame, embarrasment, fear and anxiety – may ensue independently of the use of data for nefarious purposes. However, data may certainly also be used for nefarious purposes. As in the infamous Cambridge Analytica case, it may be used for the purpose of manipulating voting behaviour through microtargeting of individuals [35]. Secondly, it is inadequate because there are other potential costs to health record research than the mere privacy breaches per se and the use of private data for nefarious purposes.

#### The costs of health record research 2: privacy, trust and adequate treatment

Privacy breaches of health records may add to existing concerns, worries and fears about the security, privacy and confidentiality of health care information. There is vast evidence of patients’ concerns, fears and worries about the security and privacy of health data in health records [36–38]. Studies indicate that a large majority ranging from 68 to 82% are concerned about the privacy and security of their health records or the exchange of health information in the health care system [39–41]. A study found that 66% of have privacy concerns in relation to sharing of health information for research [42]. In so far as there is an ‘anxiety component’ of these concerns, worries and fears towards the security and privacy of sensitive health data, it is arguably a harm in itself.

Privacy breaches may also affect trust in health care professionals and researchers. A study found that privacy concerns were negatively associated with trust in the health care system, including trust in researchers [43]. Increased privacy concerns reduce trust. Reduced trust in interpersonal relations is arguably a harm in itself – trust is of intrinsic value.

However, even if one disagrees – even if increased levels of fear and concerns about privacy and reduced trust in health care professionals and researchers are considered negligible harms in themselves – they are both closely related to a third potential harm of privacy breaches. They may lead people to withhold relevant information from health care professionals and to avoid seeking care in order to protect their privacy. Studies indicate that between 12 and 15% of patients have privacy concerns about health records to an extent that make them withhold information from health care personnel [44–46]. Several studies indicate that across different groups of patients’ concerns about confidentiality negatively affects disclosure of information and the seeking of care [38]. Self-protective behaviour of these kinds may not only have harmful consequences for individuals by ultimately causing inadequate treatment, i.e. non-maximal prevention of harm. It may also undermine the very possibility of conducting health record research by leading to incomplete and misleading data in health records.

Although there is evidence suggesting that privacy concerns are sensitive to the identifiability of the health information, the evidence also suggests that the de-identifiability of information does not annul such concerns [42, 47]. In any case, health record research will in many cases involve the use of identifiable health information (pseudonymised), and recent research suggests that in a number of cases the amount of personal information/attributes may be extensive to a level that will make an allegedly anonymous individual identifiable [48]. But even if we assume that de-identifiability may be achieved and that it may annul peoples’ privacy concerns, there are other costs of health record research that cannot be avoided by making data de-identifiable.

#### The costs of health record research 3: Denormalization by stigmatization and medicalization

Following Goffman, stigmatization occurs when a person is attributed with a discreditable trait that will make the person seem inferior, dangerous and even inhuman, and when this act of discrediting results in discrimination [49]. A recent reinterpretation [50] identifies five elements of stigmatization at a societal level: Labelling of a difference between people. Associating the identified difference with negative stereotypes. Segregation of people into groups of ‘us’ and ‘them’. Status loss and discrimination by differential treatment of the ‘labelled’ from relevantly similar groups. Asymmetrical distribution of social, economic and political power allowing for the labelling party to produce segregation and discrimination [51].

Stigmatization of people with particular types of illnesses is well evidenced. Mental illnesses is known to be associated with negative stereotypes, [52] and there is evidence of discrimination of mentally ill in the labour market as well as in health care [53, 54]. Stigmatization of the mentally ill has a number of harmful effects. It may in some cases lead to self-stigmatization – i.e. the application of negative stereotypes to oneself – which can lead to lowered self-esteem, demoralisation and self-discrimination [50, 55–57]. Widespread negative attitudes towards mental illnesses also keeps patients from seeking care, [58] and thus prejudice may lead to health inequality [59]. Studies on the effects of stigmatization of people with AIDS show that the feeling of being stigmatized is associated with anxiety, depression and distrust, and the disruption of normal social relationships [60, 61]. There is evidence of stigmatization and its harmful effects for a wide variety of groups of people, including smokers and obese people [62–66].

Note that as defined here stigmatization necessarily involves discrimination by differential treatment of relevantly similar people and groups. It seems, however, that negative stereotyping, segregation and marginalisation of groups may also be associated with other forms of differential treatment that do not qualify as discrimination but still may be considered questionable. Thus, it seems that these forms of denormalization may also lead to the stratification of solidarity. That is, it may lead to some people or groups being excluded from our help on individual and societal level due to identified, and arguably also relevant, differences between them and others. In an article in the BMJ in 1993, two surgeons posed the question “Should smokers be offered coronary bypass surgery?”, and argued that it was not justified due to the remediable cause of the need for surgery [67]. In other words, the solidarity of the health care system should be limited to cases where the need for surgery is not self-inflicted [68, 69]. If one believes that the solidarity of a health care system should be extended to people even where their needs are self-inflicted, then the suggested stratification is undesirable. Whether such suggestions – reasonable or not – are the outcome of stigmatization or perhaps the cause of stigmatization is hard to determine. The contention here is only that there are complex social phenomena such as stigmatization that are linked to different kinds of differential treatments such as discrimination and stratification of solidarity.

Can health record research – intentionally or unintentionally – stigmatize vulnerable groups of patients? A recent study showed that the health care costs to society of having a Body Mass Index (BMI) between 30 and 40 are app. € 1400 higher per year than the costs of the average person [70]. The study was based on data in various registries. These data are partly collected from electronic patient records, and in any case, there seems to be no reason to think that the difference in origin of data – whether it be registries or health records – entails a difference for the stigmatizing potential of this study. Certainly, the stigma of obesity is already in place and there was probably no intention of stigmatizing this group through the research project in question. Nonetheless, it seems that such studies may add to the stigmatization of obese people by providing new dimensions for negative stereotyping, namely the economic burden to us all of obesity. Is it unthinkable that such research will fuel already existing discrimination of obese people and drive a public or political demand for stratification of the solidarity towards obese people where the obesity is self-inflicted?

There is a second way in which health record research may have a denormalizing effect and that is through medicalization. The notion of medicalization was introduced by Zola to describe the process by which everyday activities come to be described in medical terms [71, 72]. In so doing these activities become potential targets of medical intervention, and medicine has in this way expanded its power as an institution exercising social control [71, 73]. A closely related notion of medicalization ties it to the tendency of people to view everyday activities from the perspective of medicine. Medicalization is the process whereby people change their interpretation of activities from something that is described in non-medical terms to something described in medical terms [72, 74]. Medicalisation in this latter sense raises at least three problems. First, it may generate new concerns and worries. Looking at everyday activities from within the perspective of medicine involves assessing the contribution of these activities to one’s health. Identified threats may generate concerns and worries and thus negatively impact quality of life [72]. Recent research shows that people suffering from health anxiety are at increased risk of ischaemic heart disease [75]. The harm of causing anxiety cannot be limited to psychological effects. Secondly, it changes the meaning and value of activities, and this change may be considered a loss. If, for instance, certain social activities come to be valued primarily for their contribution to health rather than for their relationship value, then this very change in meaning and value may be considered a loss. Thirdly, an imposed change in meaning may also lead to self-imposed changes of and limitations on behaviour – changes and limitations of autonomy – that are ultimately unwanted but a consequence of the imposed change in meaning of events.

Can health record research – intentionally or unintentionally – lead to medicalization? The study on the costs of a BMI introduced above may provide a convenient example. The study showed an increased annual cost to health care of having a BMI between 30 and 40. In short, a high BMI is associated with an increased need for treatment. The study in this way construes a BMI as medical condition or at least as a risk factor of a medical condition. In so doing the study underpins an already existing medicalization of high BMI. Thus, in 2013 obesity (BMI > 30) was recognised as a diagnosis by the American Medical Association [76]. There may good reasons for doing so, but the construal of high BMI as a medical condition may also have some undesirable consequences for our interpretation of everyday eating. Thus, the social or pleasurable function of eating may give way to a more health focused interpretation of eating, where the eating is mainly seen in light of its contribution to a good health. Worries and unreasonable self-imposed restrictions on behaviour may ensue.

Health record research may – as health care research in general – to varying degree drive stigmatization and medicalisation. Some would perhaps claim that it may only to a limited degree add to such harms. Three things should be noted, however. Firstly, that it may be difficult to predict *exactly* when and to what extent such harms will ensue since they depend on various personal and social conditions – they are in the broadest possible sense situational. Therefore, it is difficult to identify those health record research projects that cause no or limited harms related to stigmatisation or medicalisation and those which cause greater harms of those kinds. Secondly, it must be noted that these harms are independent of the anonymity and security of data, and thus the mere fact of conducting a health record research project may provoke such harms, even when there are not privacy breaches. They are phenomena that occur in an intricate interplay between public behaviour, societal institutions such as the health-care system and research institutions and the individual. Thirdly, it should be noted that even if we assume that a given health care research project will add only little to the stigmatization and medicalisation of a certain group of people, and that the probability of doing so is low, the negative effects of this denormalization are, as outlined above, severe. The risk of harm is a combination of all of these factors. (See also ‘Minimal or comparable harm’ below for further discussion).

#### The costs of health record research 4: unequal access to medicine

Research may also add to existing inequality in access to health care and medicine. In 2004 WHO concluded that almost 2 billion people did not have access to essential medicines, and that in some of the lowest income countries in Africa and Asia less than half the population has regular access to medicine [77].

Health research is claimed to drive inequality in access to medicine in two ways [78, 79]. Firstly, by the pharmaceutical industry allocating only very limited resources to research into medicine for diseases primarily found among the poor. In 2000 an estimated 10% of the global spending in both public and private sector on health research was allocated research into diseases or conditions that account for 90% of the global disease burden [80–82]. The so-called 10/90 gap. In the 30 year period from 1975 to 2004 1556 new drugs were approved [83, 84]. Of these 10 were for the most neglected tropical diseases, 8 were for malaria and 3 for tuberculosis. Malaria and tuberculosis account for more than 5% of the global disease burden [82]. Secondly, by the pharmaceutical industry prizing medicine much higher than the costs of production, i.e. by maximising profits. The patent system gives the pharmaceutical companies the exclusive right to produce and distribute medicine. In a normal competitive market, the availability of substitute medicine will restrain the companies’ prizing [85]. However, if there are no substitutes for a given drug, a patent may create a monopoly and this will typically lead to higher prizes. In countries with significant inequality in income and wealth, a monopolist pharmaceutical company will maximise profits by selling its products at a prize that only few can afford. For instance, a monopolist company producing antiretrovirals for people with AIDS in South Africa will maximise profits by selling at a prize that only the top 10% can afford [85]. There is evidence of such prizing differences between developing and developed countries [85–87]. Public health care can absorb such differences in prizing, but in many countries – and especially in developing countries – health care is largely financed out-of-pocket [87].

Health record research may – as all kinds of health care research – in many different ways feed in to the pharmaceutical research and development of medicine, and thus also preserve and further existing inequality in access to medicine.

#### Minimal or comparable harm?

Mann et al. argue that the risk of health record research is privacy breaches where data are used for nefarious purposes, and this risk satisfies different standards of minimal harm. What has been argued here is that there are more potential harms of such research. All of these possible harms must be further investigated.

The question is, if these costs or harms are minimal? Or, as Singer would put it, if they are comparable to the benefits of contributing to research? The view taken here is that the potential harms of conducting health care research including research into electronic patient records cannot be considered *minimal* if it, among others, may lead to patients withholding information and consequently receiving suboptimal treatment, if it may produce or add to stigmatization, discrimination and increased stratification of solidarity, and if it may preserve and further existing inequality in access to vital health care. Whether or not such harms are *comparable* depends on the very notion of comparability. Singer speaks of something being of “comparable moral importance”. It seems that the potential harms listed here are of comparable moral significance exactly in the sense that they make it morally permissible not to contribute to such research. They may not at all be sufficient to outweigh our reasons for conducting health record research, but they may be sufficient reason to annul a duty of easy rescue to participate in such research.

It may be objected here that the mere possibility of significant harms cannot be sufficient to annul our duty to contribute to health record research just as the mere possibility of the drowning child later becoming a serial killer cannot annul our duty to save him or her from drowning. The latter is certainly true. There is, however, a relevant difference between the potential harmful effects of contributing to health record research and saving the drowning child. In the case of health record research, the harms are somewhat predictable, and thus somewhat avoidable. There are underlying causal mechanisms that we to some extent may detect and influence. We may as potential health record research participants form qualified opinions about whether a research project requires too much or too sensitive information, whether privacy is sufficiently well protected, whether there is a risk of abuse of personal and sensitive information, whether there is a risk of certain groups being further stigmatised or ordinary life practices becoming medicalised, whether the research project is yet another case of research into ‘the white man’s diseases’ and so on. And, having detected and predicted that a health record research project is more or less likely to have harmful effects, we may encourage the researchers to halt or redesign the project, stir public debate on research of this kind and potentially withhold/withdraw consent and encourage others to do the same. Ultimately, it is the significance of the potential harms and their predictability (/unpredictability) in combination with the unpredictability (/predictability) of the benefits that is argued to annul a Singer style duty of easy rescue to contribute to health record research.

### Greenspan’s duty of easy rescue and health record research

Moving to the opposite end of the spectrum of duties of easy rescue, does not make a difference. In Greenspan’s view the duty of easy rescue is a duty to rescue the victims of accident we encounter in our daily lives. If participation in health care research should be entailed by a duty of easy rescue, it must be directed at these people accordingly. However, health care research is not an instrument for rescuing sudden victims of accident. It is rarely, if ever, directed at rescuing people struck by sudden disaster. Given the time-consuming nature of conducting good research and the time-span from scientific discovery to actual health care (see above), health care research will most often be directed at preventing harm to future, potential patients. Hence, in Greenspan’s interpretation an individual’s duty of easy rescue would not entail a duty to participate in health care research.

But what about the collective duty of easy rescue – the state’s duty of easy rescue? Does that entail a duty to conduct research, and if so do citizens then have a derivative duty to participate in health care research and research on electronic patient records in particular? A state with a significant – perhaps global – outreach would be faced with the problem of a Singer style duty of easy rescue. It would have to prioritise between different efforts of rescue. Greenspan’s notion of the state’s duty of rescue does not have any implications for how to prioritise between different ways of providing rescue. It is an open question whether the state should give priority to health care research over famine relief.

There are, however, reasons to think that the state should set up and support a system for health care research. By setting up and supporting various different types of societal activities aimed at preventing harm, including various types of health care research, the state ensures a certain spread in its efforts of preventing harm and it solves a coordination problem for those of its citizens trying to prevent harm through health care research. By spreading and coordinating the efforts, the state may increase the chances of maximising the prevention of harm. Furthermore, by spreading its activities the state may achieve equality in more dimensions, e.g. equality in health outcomes. But if so, would citizens then have a derivative duty to contribute to all possible state-sanctioned efforts and activities on Greenspan’s account? Would citizens have a duty to contribute to health care research, and health record research in particular?

There is conflict here between the autonomy granted in the imperfect duty to aid and care – our ‘moral leeway’ – and the state paternalism justified by the state’s duty of rescue. The state may completely transform the imperfect duty of aid and care into perfect duties derived from its own duty of easy rescue. Greenspan is well aware of this conflict. As outlined above, she notes that in passing our imperfect duty of aid and care onto the state, we may have to relinquish some of our ‘moral leeway’ in order for the state to satisfy its duty of rescue. She resolves the conflict by limiting the state’s power to that of controlling the size of our contributions and the beneficiaries of these contributions. The state cannot decide the purposes to which its citizen must contribute. The citizens must decide. In this way the primacy of the citizens’ autonomy in satisfying their imperfect duty of aid and care is maintained, while the state at the same time is given a room of manoeuvrability in satisfying its duty of rescue.

How should the citizens decide the purposes of the state’s rescuing activities to which they are required to contribute? Greenspan does not address this question, but such decisions could arguably in many cases legitimately be made indirectly by democratically elected politicians. Greenspan notes, however, that it must be made by the individual citizen in cases, where the contributions required by the state is of a certain nature. Thus, if citizens are enlisted for national service, they must be given a choice between different types of service and not just military service. And, relatedly, that requiring labour and other non-monetary contributions raise harder issues of autonomy than monetary contributions such as tax. But what is the difference?

There seems to be two important, interrelated differences between monetary contributions in the form of tax and non-monetary contributions such as health record data. Firstly, money is *essentially* impersonal. It does not in itself reveal anything about its owner, and it does not make a person identifiable. On the contrary, health record data are essentially personal. They provide information about a specific person, and this information is generally considered sensitive. They make a person identifiable. Second and relatedly, money is in itself unspecific as to its use. It will generally have to made relevant for a specific purpose by virtue of its purchasing power. If money is used for research, it will have to be made relevant through funding of research. The exact role – how it will become relevant for and influence e.g. treatment or research – is highly uncertain. Not least because it, within the context of modern democracies, will depend on political decision-making. Although health data may be used for many different purposes, they are not as abstracted from potential purposes as money. Moreover, health record data are often directly relevant for their purpose. If health record data are used for research, they are of direct relevance for the outcome of that research. The upshot of these differences is this. By paying tax to the state the individual citizen is making an impersonal contribution in the sense that it may be used for various purposes, and if and how it will be used for e.g. research is fundamentally uncertain. The causal link between the citizens’ contribution and the outcome of that tax payment is very indirect and unpredictable. By providing health record data, the individual citizen is making a personal contribution that is limited in its use for more specific purposes, and the contribution is directly relevant for those purposes. The causal link between the citizens’ contribution of health record data and an outcome is less indirect and more predictable.

This difference is ethically relevant. There are at least two interrelated issues of autonomy here. First, the moral responsibility is different in the two cases. If someone makes a personal contribution and is more directly linked to the outcome of their contribution and the outcome is more predictable, then they are arguably also more responsible for the outcome. Thus, it is arguably more reasonable to ascribe greater moral responsibility to a supporter of terrorism, if he or she carries explosives to be used for terrorism than if making a financial contribution to an organisation known to be supporting terrorism. Second, the act of self-expression is different in the two cases. Making a personal contribution with a more predictable outcome is a stronger act of self-expression and commitment to a cause than merely making a monetary contribution with uncertain outcome. For these reasons it is reasonable to make a distinction between monetary contributions in the form of tax and non-monetary contributions such as health record data. The legitimate purpose of using health record data should be decided by the individual.

In conclusion, Greenspan’s model implies that neither an individual nor the state has a duty of easy rescue to do health record research. It has been argued, however, that there are good grounds for believing that the state should set up and support a system of health care research, including health record research, but also that the citizens do not have a derivative duty to contribute their health data. They may choose to do so in acting on their imperfect duty of aid and care. They may choose to do so voluntarily.

### Easy rescue, consent bias and mandatory participation in health record research

The possibility of consent bias is often used – also by Mann et al. – as an argument in favour of giving up consent requirements for health record research. In arguing for lifting the requirement of informed consent for health record research they write: “Requiring consent will lead to distorted and sometimes completely fallacious results, which, in turn, lead to death and disease that could have been easily avoided”. In short, participation in health record research must be mandatory, i.e. without informed consent, in order to maximise the prevention of harm.

Let us define consent bias the following way: Consent bias occurs if and only if the research participants consenting to research participation are not representative of the studied population and this biases the results, i.e. produces false or misleading results. Mann et al’s argument is simply that if the results of such research is applied in health care it may eventually cause harm, i.e. it may lead to non-maximal prevention of harm. There are examples of consent requirements producing biased results [88, 89]. There are also examples of consent requirements not biasing results [90, 91]. For the sake of argument, we shall simply assume that in the course of time consent requirements will inevitably come to produce consent bias, and that occasionally this will lead to the non-maximal prevention of harm by the health care system. Does that entail that participation in health record research should be mandatory?

The mere possibility of consent bias does not ground a moral obligation to participate in research, and in health record research in particular. It simply cannot establish a duty to participate. At most, it provides a moral reason to prefer mandatory participation in health records research over participation based on informed consent. At most, it shows that research participation should be mandatory *if* we already have a duty to participate. We have previously argued that on different accounts of the duty of easy rescue – Singer’s and Greenspan’s – there is no perfect duty to participate in health record research. But if a citizen duty to participate in health record research cannot be established, then the possibility of consent bias becomes ethically impotent.

### Limits of arguments from consent bias

However, let us for the sake of argument assume the existence of a duty to participate in health record research. Does consent bias then provide a strong reason for making this participation mandatory, i.e. without informed consent? In order for consent bias to be a strong reason for mandatory research participation, at least two things must be shown. First, that consent bias is a widespread and significant problem in everyday health care research, or that it is particularly likely to obtain in relation to a particular research project. Second, that the occurrence of consent bias generally or in relation to a particular research project is likely to find its way into clinical practice and cause significant harm. The mere possibility of the occurrence of consent bias cannot outweigh our strong reasons for maintaining the voluntariness of research participation (see below).

Listing as Mann et al. cases of research where consent requirements have reduced the sample size or providing evidence of differences in key characteristics between consenters and non-consenters, e.g. age, sex, race, education, income and health status, will not do the job [89]. Firstly, it is not evidence of consent bias. Although reduced sample size and difference in characteristics are prerequisites of consent bias, they do not necessarily produce consent bias. Secondly, although it may be taken to support a case for the existence of consent bias, it does not show that consent bias is widespread and significant problem in everyday health care research. It does not show that consent bias will generally apply to all research conducted on the basis of consent requirements. When defining consent bias above we provided evidence to the effect that it does not always apply. Thirdly, it does not show that consent bias will always have any real-life, harmful effects in a specific project. As previously argued it is uncertain whether a piece of research – consent biased or not – will ever make a difference for actual health care. And, even if consent biased research should make it into actual health care, the bias may be too insignificant to produce any real harm. Fourthly, consent bias may to some extent be statistically adjusted for [92, 93].

In sum, there are many uncertainties surrounding claims about consent bias in relation to a specific research project. Uncertainties, which seriously limit the relevance and potency of such arguments.

## Conclusion

This article has argued that it is *not* the case 1) that a duty of easy rescue implies a perfect duty to participate in health record research, 2) that there are only insignificant harms associated with such research, and 3) that consent bias provide a strong reason for making participation in health record research mandatory. On the very contrary, the analysis has provided ample reasons for insisting on the voluntariness of research participation, i.e. to maintain a requirement of informed consent for research participation.

Informed consent allows individuals to assess, weigh and protect themselves against the harms associated with health record research, including in particular the fears and worries concerning loss of privacy, stigmatization, medicalization, and interpersonal distrust and all of the harms that may ensue from each of these harms. Informed consent also allows for individuals to act on their values and interests. Individuals may have all sorts of values and interests of relevance for health record research, including in particular to promote research in order to benefit themselves and others and to promote altruism, but also to avoid marginalisation of certain groups or to promote a research infrastructure and organisation that does not further stratification of solidarity or inequality in access to treatment and medicine on a global level.

This protection of individuals’ wider values and interests of relevance for research cannot be done by anyone else than the individual research participant. Thus, the entire set of values and interests are truly individual, and how they are balanced in the assesment of the risks and benefits associated with research participation is truly individual – not least because we are not always consistent and unbiased in our preference-formation [94, 95]. The individuality of balancing risks and benefits has two important implications. First, it implies that research ethics committees assesments of risks and benefits cannot replace individuals’ assessments. Research ethics committees simply do not have access to the wider values and interests at individual level. Second, and relatedly, it also implies that it does not make a difference whether we look at health record research as such or consider research projects at individual level. Each research project may indeed be claimed to have its own specific risk-benefit profile, i.e. it may be claimed to be associated with smaller or greater risks and benefits than other projects. These are and must be smaller or greater as seen from the indviduals’ perspective – not through the lens of a research ethics committee.

Having dismissed in the introduction the wider discussion of consent models, it may be noted here that the possibility that individuals may believe some research projects to be ‘harmless’ and others to be ‘very harmful’ could be taken to support consent models that allow individuals to express such beliefs in their consent behaviour. Thus, a tiered consent model that would give research participants a sovereign choice between providing consent to broad categories of research or providing consent at the level of specific research projects, would provide the research participants with the opportunity to express their beliefs concerning the harmfulness of the research. If certain types of research are believed to be harmless, this could be expressed by providing broad consent to these types. If, on the contrary, other kinds of research are believed to be more harmful, this could be expressed by the research participants requiring to provide consent at the level of the specific research project. This is one of the essential ideas of the meta consent model [16, 19]. For present purposes it should simply be noted, however, that if it is the individual research participant, and not research ethics committees, that ultimately should determine whether research is harmless or harmful, then we should look for ways in which individual, and not research ethics comittees, can express such views. From this perspective, empowering research ethics committees with the authority to grant exemptions from requirements of informed consent to health records research is simply the wrong solution, and as shown in previous sections it lacks firm ethical basis.

This is obviously not to say that research ethics committees are redundant. What has been argued so far is that informed consent is a necessary ingredient in the protection of individual research participants. In actual practice, it may turn out to be an insufficient instrument for the protection of individuals against harms and for protecting individual autonomy in a wider sense. Individuals may for various reasons fail to protect themselves against basic forms of harm that are evident harms for a research ethics committee. Protection against harms identified by a research ethics committee may turn out to be far superior to no or very poor self-protection.

Finally, there is a noteworthy and non-negligible effect of mandatory research. Thus, as many writers have observed there seems to be an intimate link between trust and informed consent [96–100]. The provision of truthful and adequate information and asking for consent ceteris paribus builds trust. Deception and coercion undermine trust. The processes involved in obtaining informed consent – providing truthful information and involvement of individuals in decision-making – certainly can be argued to provide good reasons for trusting someone. However, whether or not informed consent processes actually produce the psychological phenomenon of trust is ultimately an empirical question. An extensive qualitative and quantitative study of the American public’s attitudes towards informed consent in relation to the use of biological samples in a national biobank showed that 75% agreed or strongly agreed that informed consent for every specific use of biological samples would lead them to have more trust [101]. Trust is undoubtedly a complex phenomenon with many situational variables, but the relevant study suggests that making mandatory the provision of data for health record research may affect trust in researchers and research institutions negatively. In this sense, one could argue that contributing to the potential decline in public trust is an even greater threat to health care research than consent bias.

## Data Availability

Not applicable.
